# Model Reconstruction from Small-Angle X-Ray Scattering Data Using Deep Learning Methods

**DOI:** 10.1016/j.isci.2020.100906

**Published:** 2020-02-13

**Authors:** Hao He, Can Liu, Haiguang Liu

**Affiliations:** 1 Complex Systems Division, Beijing Computational Science Research Center, 8 E Xibeiwang Road, Haidian, Beijing 100193, People's Republic of China; 2 School of Software Engineering, University of Science and Technology China, Suzhou, Jiang Su 215123, People's Republic of China; 3 Physics Department, Beijing Normal University, Haidian, Beijing 100875, People's Republic of China

**Keywords:** Computational Molecular Modeling, Algorithms, Computer Science Applications

## Abstract

Small-angle X-ray scattering (SAXS) method is widely used in investigating protein structures in solution, but high-quality 3D model reconstructions are challenging. We present a new algorithm based on a deep learning method for model reconstruction from SAXS data. An auto-encoder for protein 3D models was trained to compress 3D shape information into vectors of a 200-dimensional latent space, and the vectors are optimized using genetic algorithms to build 3D models that are consistent with the scattering data. The program has been tested with experimental SAXS data, demonstrating the capacity and robustness of accurate model reconstruction. Furthermore, the model size information can be optimized using this algorithm, enhancing the automation in model reconstruction directly from SAXS data. The program was implemented using Python with the TensorFlow framework, with source code and webserver available from http://liulab.csrc.ac.cn/decodeSAXS.

## Highlights

• A convolutional neural network auto-encoder framework for 3D models is developed
• The auto-encoder compresses protein shape information to 200 parameters
• Accurate 3D models (both shape and radius) can be reconstructed from 1D SAXS data

## Graphical Abstract

**Figure:**
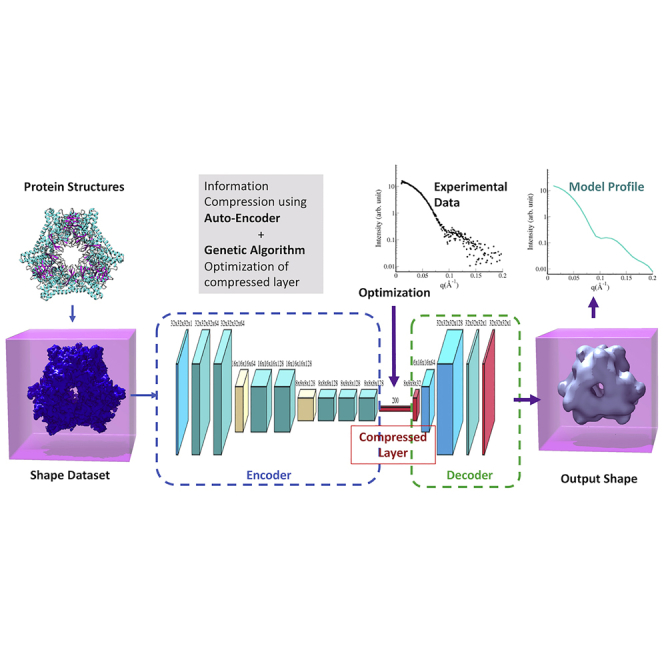

## Introduction

Small-angle X-ray scattering (SAXS) from protein molecules in solution is a powerful technique that provides information on molecular structures and dynamics (Grant et al., 2011, Putnam et al., 2007, Svergun and Koch, 2003). Because the solution scattering method does not require special treatment for protein molecules, such as crystallization in diffraction measurement or isotope labeling in nuclear magnetic resonance, SAXS experiments can be performed in high-throughput manners (Hura et al., 2009). Another major advantage of SAXS experiments is the ability to probe the structure and dynamics in solution, which is especially useful when combined with pumping methods to promote conformational changes (Kim et al., 2012, Neutze and Moffat, 2012). Time-resolved studies will reveal important information on molecular mechanism for protein functions.

Despite the success in extracting structural information from SAXS profiles, reconstructing high-quality 3D models remains challenging. Several approaches have been proposed and implemented to build 3D density maps from SAXS data, including shape envelope approximation using spherical harmonics functions, polymer chain folding, dummy atom assembly, iterative phasing, and database searching methods. The spherical harmonics function approximation method is fast but limited by resolution (Stuhrmann, 1970, Svergun and Stuhrmann, 1991, Svergun et al., 1996). In the Gasbor program, polymers composed of connected beads were used to represent protein molecules, and the packing of these polymers was optimized to build 3D models (Svergun et al., 2001). Dummy atoms arranged in a 3D lattice were also used for model reconstruction, as implemented in DAMMIN/DAMMIF (Franke and Svergun, 2009, Svergun, 1999). An iterative phase retrieval method was expanded to analyze SAXS data and demonstrated its applications (Grant, 2018). Using machine learning methods, Franke et al. developed a method to classify the shapes and gain valuable model parameters (Franke et al., 2018). There are also successful attempts to integrate SAXS data to molecular prediction/modeling/simulation frameworks to obtain 3D structures (Hura et al., 2019, Karczyńska et al., 2018). A database of shapes abstracted from actual protein complexes and efficiently represented using 3D Zernike polynomials was used to quickly retrieve 3D models that match experimental SAXS profiles, as implemented in sastbx.shapeup (Liu et al., 2012b). A real space representation of a 3D model requires many parameters, such as the position of each bead, which can be described using its coordinates (then 3N parameters are required for a model with N beads). However, the number of parameters required is much greater than the number of free parameters encoded in 1D SAXS profiles (Moore, 1980). Therefore, prior knowledge must be applied to provide additional constraints for converged reconstructions. For example, the molecular size and the connectivity of the beads are very critical for DAMMIN/DAMMIF. In the case of sastbx.shapeup, the molecular size is de-coupled from the abstracted shapes (Liu et al., 2012a), allowing an optimization of the size as a separate parameter. However, the diversity of models is limited by the database. Model reconstruction will be significantly advanced if the following criteria are met: (1) diverse shapes of 3D models can be efficiently represented to cover a broader range than those in structure databases and (2) SAXS profiles can be computed for each model that can be scaled to arbitrary sizes. We provide a solution to achieve this using an auto-encoder method combined with 3D Zernike representations (Canterakis, 1999, Liu et al., 2012a).

**Figure 1:**
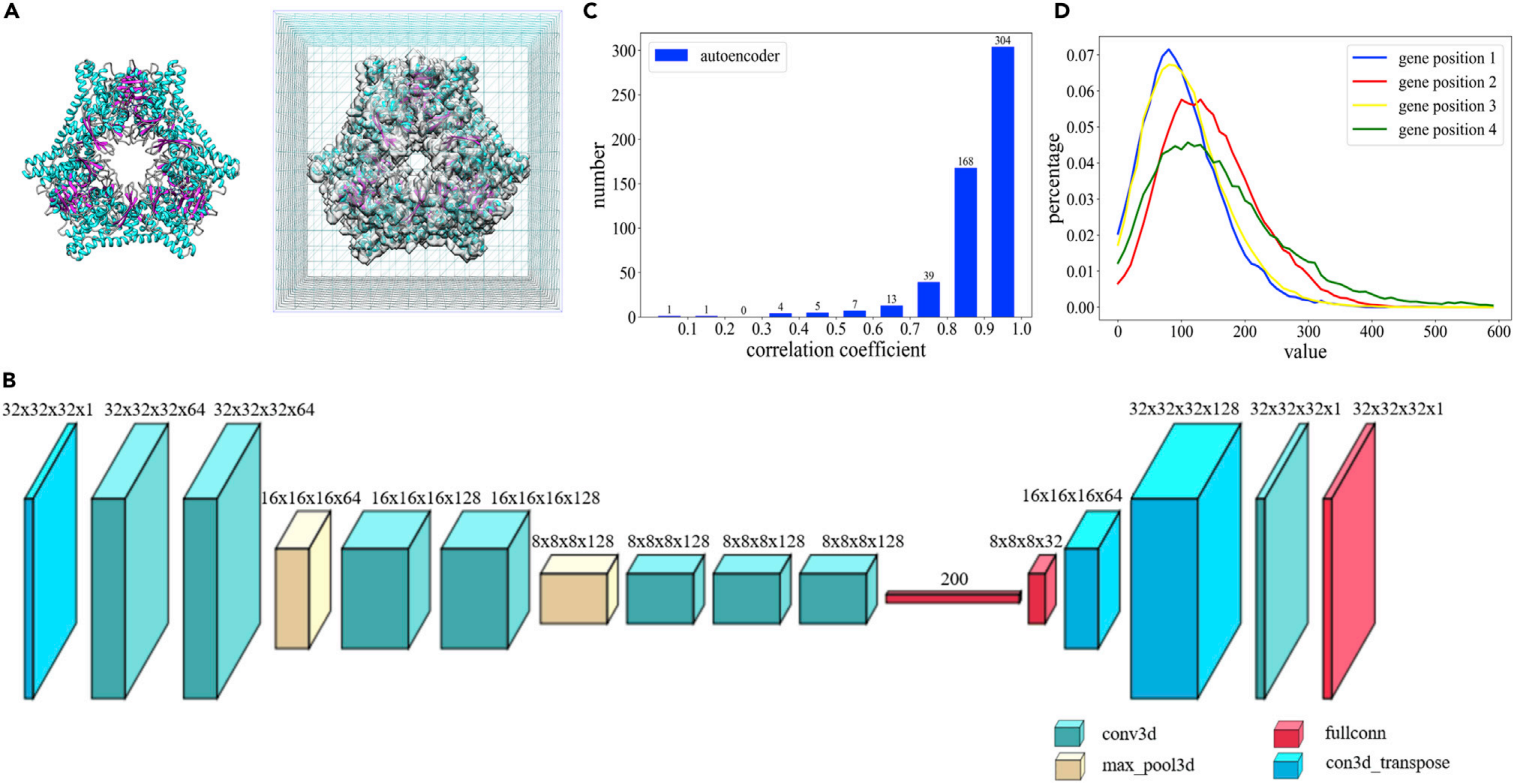
Framework of Auto-Encoder and Its Capability in Representing 3D Models (A) The voxelization of a molecular structure. Left: an atomic model represented in the cartoon representation. Right: the model is mapped on a 3D matrix whose values are binarized depending on whether the grids are in the vicinity of any atom of the protein molecule. (B) The auto-encoder-decoder architecture used in this study. The layers and structures are shown in the figure; details can be found in the Transparent Methods. (C) The model quality encoded using the trained network and measured using the correlation coefficients between the models before and after going through the encode-decode process. (D) The distribution of encoding parameter values for first four parameters in latent space.

Inspired by deep learning methods, real space 3D models were encoded using an auto-encoder neural network to a compressed representation of 200 latent parameters. The protein complexes in the PISA database (Krissinel and Henrick, 2007) were used to generate the training datasets for the auto-encoder. Each complex structure was scaled to the same radius (50Å was used in this study) then voxelized on a 3D grid of 31 × 31 × 31 (i.e., voxel edge size is 50/15Å). Because SAXS data often provide low-resolution information that warrants a uniform density approximation for 3D models, we binarized the voxelized objects before auto-encoder training. Owing to the uniform density approximation of the reconstructed models, the SAXS data comparison were limited up to q = 0.2 Å^−1^ (Grant, 2018, Poitevin et al., 2011). The auto-encoder model is based on the VGG network (Simonyan and Zisserman, 2015), composed of convolutional, pooling, and full-connected layers (see Figure 1, Table S1 and the Transparent Methods). The testing results show that diverse shapes represented using 31^3^ voxels with binary numbers can be accurately encoded using a vector of 200 dimensions. The reduction of parameter space allows application of optimization algorithms to improve the model-data fitting. SAXS profiles for 3D voxelized objects were computed using the Zernike expansion method, taking advantage of fast evaluation of theoretical profiles at an arbitrary model radius. The genetic algorithm (Goldberg, 1989) was used to optimize the latent space parameters, which were decoded to 3D models subsequently. One of the major advantages of the proposed method is the automatic determination of the model radius, which can be coded as an additional parameter and subject to the optimization along with the other 200 parameters. The testing results using experimental data from the SASBDB (Valentini et al., 2015) and BIOISIS (Rambo and Tainer, 2011) show that the proposed method can successfully generate 3D models based on SAXS data. The algorithm is implemented to the software, *decodeSAXS*, whose source code and an associated webserver are available at http://liulab.csrc.ac.cn/decodeSAXS.

## Results

In this section, we first demonstrate that the auto-encoder neural network accurately represents the shape information in the compressed form. Then, we show the performance of model reconstruction with or without model size information using the SAXS data as the target for optimization. The performance was compared with other widely used model reconstruction algorithms. The model quality and consistency were also assessed by running multiple reconstructions and analyzing the similarity among reconstructed models. The reconstructions for experimental SAXS datasets yield high-quality 3D models in general with a few exceptions for challenging cases, such as loosely packed molecules or those with large cavities.

### Quality and Accuracy of 3D Model Auto-Encoding

The voxelized objects derived from protein complex structures were encoded using 200 latent parameters in the auto-encoder neural network training procedure as described in the Transparent Methods. From two databases for small-angle scattering, the SASBDB and the BIOISIS, 542 SAXS datasets with deposited 3D models were obtained (see Supplemental Information for the full list). First, using the 3D models from these 542 datasets, the auto-encoder performance was evaluated. Each model was converted to a 3D voxel object with binary values and then fed to the trained auto-encoder for encoding and decoding. Then, each input model was used as the reference to assess the quality of the decoded 3D object. The real space correlation coefficient (denoted as *cc*, see Transparent Methods for detailed explanation) between original models and decoded models from the corresponding 200 latent parameters were computed and analyzed. The correlation coefficient is used to quantify the fraction of the overlapped volume compared with the geometrically averaged volume of two models. The auto-encoder network is very efficient and accurate in representing the majority of the 3D protein shapes, yielding a mean *cc* of 0.88, with 304 (56.1%) models having *cc* values greater than 0.90 (see Figure 1C). We also observed a few failed cases, whose correlation coefficients are very low. After investigating those failed encoding cases, we found that those models have very complex shapes, such as flexible chains (for instance SASDBD:SASDBZ6, with *cc* = 0.40) that have multiple conformations in the deposited dataset. For the majority of the testing models that have compact shapes, the auto-encoder works nicely in representing 3D shape information. The encoding-decoding test demonstrates that the 3D models are accurately reproduced after going through the encoding-decoding procedure, and the compressed 200-d vectors are sufficient to represent 3D molecular shapes. This lays the foundation for applying the auto-encoder method to reconstruct 3D models by optimizing compressed parameters to obtain models that fit to experimental SAXS data. Furthermore, the training dataset provides the distributions of latent variables, indicating that the valid values for these variables are distributed in limited ranges (see Figure 1D for distributions of representative variables). This prior information will facilitate the parameter sampling for the gene pool initialization and during the optimization process using genetic algorithms (see Figure S1 in Transparent Methods).

### Performance of Reconstruction Algorithm with or without Model Size Information

The evaluation of the reconstruction algorithm was performed on the same 542 experimental datasets that were used to evaluate the performance of the auto-encoder neural network in the previous section. Here, we use the SAXS data as the only information to reconstruct the corresponding 3D models, and the deposited 3D structures along with the SAXS data are used as the references for model quality assessment after reconstruction (see Table S4).

The first reconstruction experiment was performed by assuming that the model size information is known. Model sizes can be derived from SAXS data using GNOM or other similar approaches (Liu and Zwart, 2012, Svergun, 1992). Instead of the maximum dimension obtained directly from the pairwise distance distribution functions, the auto-encoder method used the radius of minimal bounding sphere for the desired model (hereafter referred to as model radius). Here, the radius of deposited models (coarse-grained bead models or atomic models) from each SAXS dataset was used as the input radius for model reconstruction.

**Figure 2:**
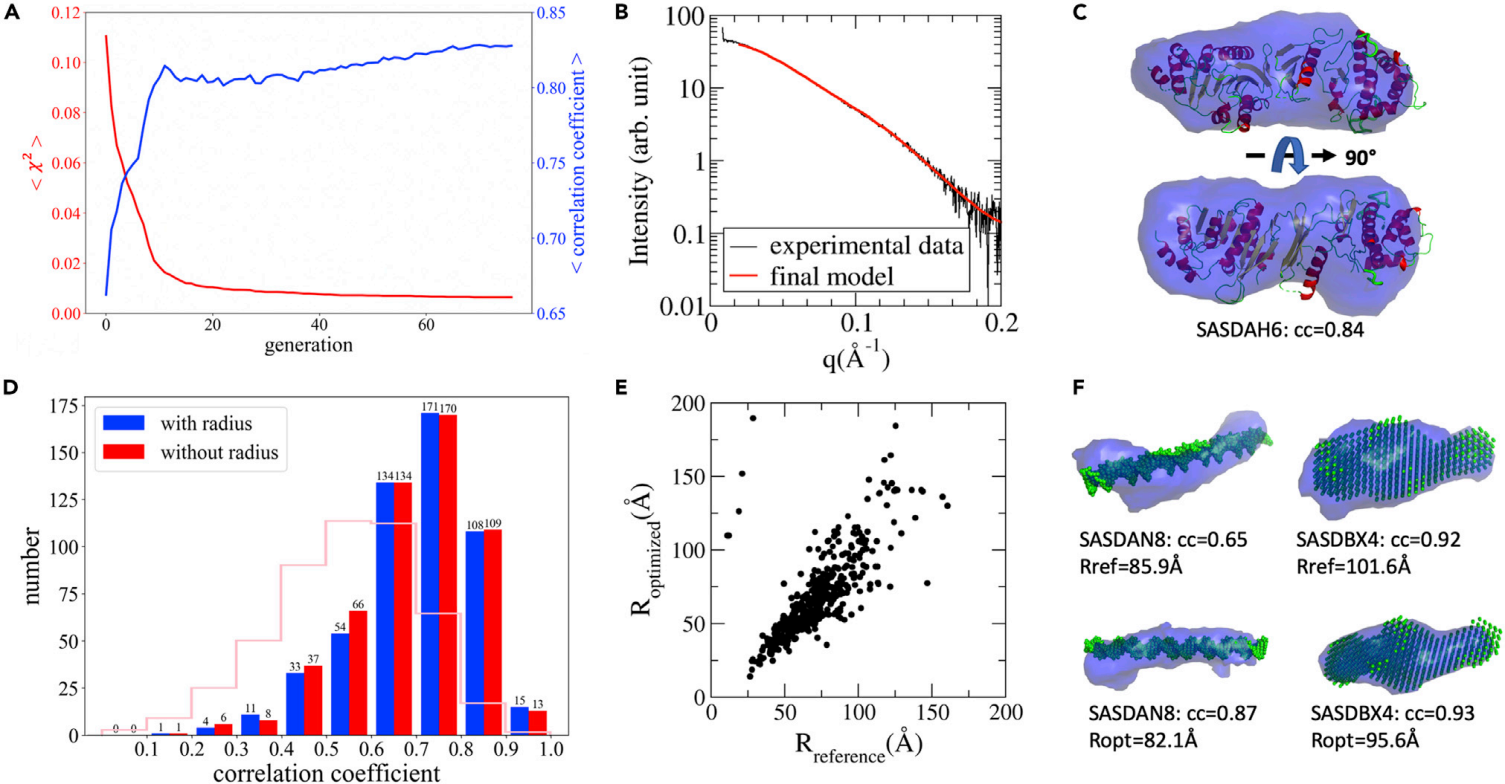
Performance of the *decodeSAXS* Algorithm (A) The progress of model reconstruction by optimizing the goodness of fit to experimental data (SASBDB:SASDAH6). Chi-scores for SAXS data and the correlation coefficients for reconstructed models are shown for each iteration. (B) The SAXS comparison with experimental data for the reconstructed model. (C) The reconstructed model (blue surface) compared with the reference structure (obtained from SASBDB) at two orthogonal orientations. (D) The algorithm performance measured using correlation coefficients between reconstructed models and the reference structures in the databases. Blue and red histograms show the statistics of correlation coefficients with or without using model size information as prior knowledge, respectively. The histogram represented with pink lines indicates the correlation coefficients for random paired models. (E) The comparison between optimized model radii and the reference model radii. (F) Representative reconstructions for two examples from the SASBDB with or without radius information.

The reconstruction process was monitored based on the chi-score between model SAXS profile and the experimental data. As a retrospective check, the reconstructed models are also compared with the reference model by computing their correlation coefficients after optimal alignment. Figures 2A–2C present an example to demonstrate the progress of model reconstruction. The dataset is from the SASBDB (SASDBD:SASDAH6), and the atomic structure deposited along with the SAXS data was used for model quality assessment. The chi-scores of SAXS data comparison and the *cc* from the 3D model comparison are shown for each iteration. As shown in Figure 2A, the chi-score was rapidly reduced within the first 10 iterations and gradually converged to a small value, indicating that the model SAXS profiles match the target SAXS data. Meanwhile, the correlation coefficients were improved as the model was iteratively reconstructed. The final model SAXS profile is shown in Figure 2B compared with the experimental data. The reconstructed model was superimposed onto the atomic model and shown in Figure 2C in two orthogonal orientations. The agreement between the reconstructed model (blue surface) and the atomic structure (cartoon model) illustrates the accuracy of the 3D model reconstruction for this dataset. The reconstruction process can also be directly visualized in real space by showing a sequence of reconstructed models in the form of videos (see Video S1 for an example). The progress clearly demonstrates that the genetic algorithm is capable of driving the model reconstruction by improving the goodness of fitting to experimental SAXS data.

Video S1. Video Demonstration of Model Reconstruction Progress as a Function of Iteration Steps, Related to Figure 2 This is for the reconstruction of SASDA38.

Figure 2D shows the statistics of reconstructed model quality measured using correlation coefficients between the reconstructed models and the reference models. The histogram colored in blue shows reconstruction performance using the model radius as known information. As shown in the following, the radius information is not required for the reconstruction algorithm to achieve similar accuracy levels. Among 542 testing datasets, 294 reconstructed models have correlation coefficients greater than 0.70 (see Table S2 for representative models at various *cc* levels for visual inspections). At this level (*cc* = 0.70), the reconstructed models are consistent with the references in the overall shapes according to visual inspections. The models with a big cavity or flexible domains are challenging for the auto-encoder to compress the shape information to a 200-dimension vector, as discussed in the previous section. For models with rigid and compact structures, the auto-encoder and the SAXS-based reconstruction are very successful. Furthermore, random pairing the models was used as the control method, and the *cc* between randomly selected models were used to estimate a baseline for reconstruction performance assessment. Prior to the alignment and correlation coefficient computation, two models randomly selected from the 542 reference models were scaled to the same size. This procedure was repeated for 100,000 times to get the statistics of the *cc* values, which are scaled and shown with pink lines in Figure 2D. The average *cc* value of the control method is 0.55 with a standard deviation of 0.16, suggesting that the expectation value of *cc* is 0.55 by random matching. The *decodeSAXS* algorithm performed much better than random matching. Among 542 reconstructed models, 86% showed *cc* > 0.55 and 54% models had *cc* > 0.70 in the case of the *decodeSAXS* using correct model radius.

**Figure 3:**
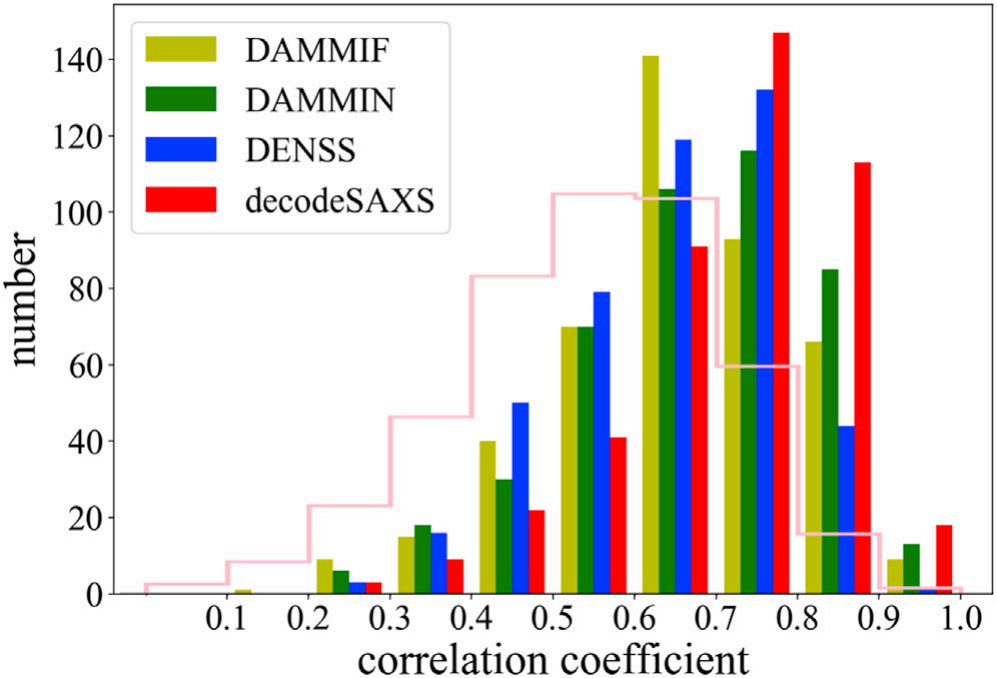
Performance Comparison with Other Methods The histograms of the correlation coefficients between reconstructed models and reference models indicate that *decodeSAXS* outperforms the other three methods. The histogram with pink color shows the statistics of correlation coefficients between randomly selected models, providing a baseline to assess the performance of reconstruction algorithms.

Three other methods, DAMMIN, DAMMIF, and DENSS, were applied to the same dataset for model reconstructions. To reduce the bias during model reconstructions, default parameters and correct radii were used for model building in all programs. The reconstructed model quality was measured using the correlation coefficients and compared with the *decodeSAXS* method (Figure 3). The results revealed that all reconstruction methods generated models better than randomly selected models, whose statistics was used as a reference (pink line in Figure 3). Detailed analysis showed that the performance of DAMMIN was better than DAMMIF in general, whereas DENSS achieved similar accuracy as DAMMIN. Based on the statistics, *decodeSAXS* program outperformed these three methods on the testing dataset, reflected by larger populations with higher *cc* values (red color histogram in Figure 3). The scattering plots of *cc* values between references models and those reconstructed using either *decodeSAXS* or the other methods are summarized in Figure S3, showing better performance of *decodeSAXS* in detailed comparisons.

In practice, the size information of the model to be reconstructed could not be accurately obtained in many cases, preventing successful model reconstructions. In such cases, the *decodeSAXS* algorithm can optimize the model size under the same framework by simply treating the model radius as an additional element in the genes (the parameters). Using the same dataset, the reconstruction algorithm was tested without providing the size information. The initial radius for each model in the first generation is a random positive number smaller than 300 (with the associated unit Å). The radius was taken as the 201st parameter and subject to the genetic algorithm for optimization. The optimized radii for 542 testing datasets are compared with the radii extracted from the reference models in Figure 2E, showing that the size information can be obtained by optimization. More importantly, the reconstructed model quality without using the correct model radius is comparable with the outcomes with radius information as shown in Figure 2D (red color histogram for the cases with optimized radius, and the blue color histogram is for the case with correct radius, whereas the pink lines show the statistics for the comparison between randomly paired models). Two examples shown in Figure 2F demonstrate that high-quality 3D models can be reconstructed even if the radius is not exactly the same as the values of reference models, indicating the robustness of the algorithm. The automated determination of model radius during reconstruction process is an important feature of the *decodeSAXS* algorithm.

### Consistency of Model Reconstructions

**Figure 4:**
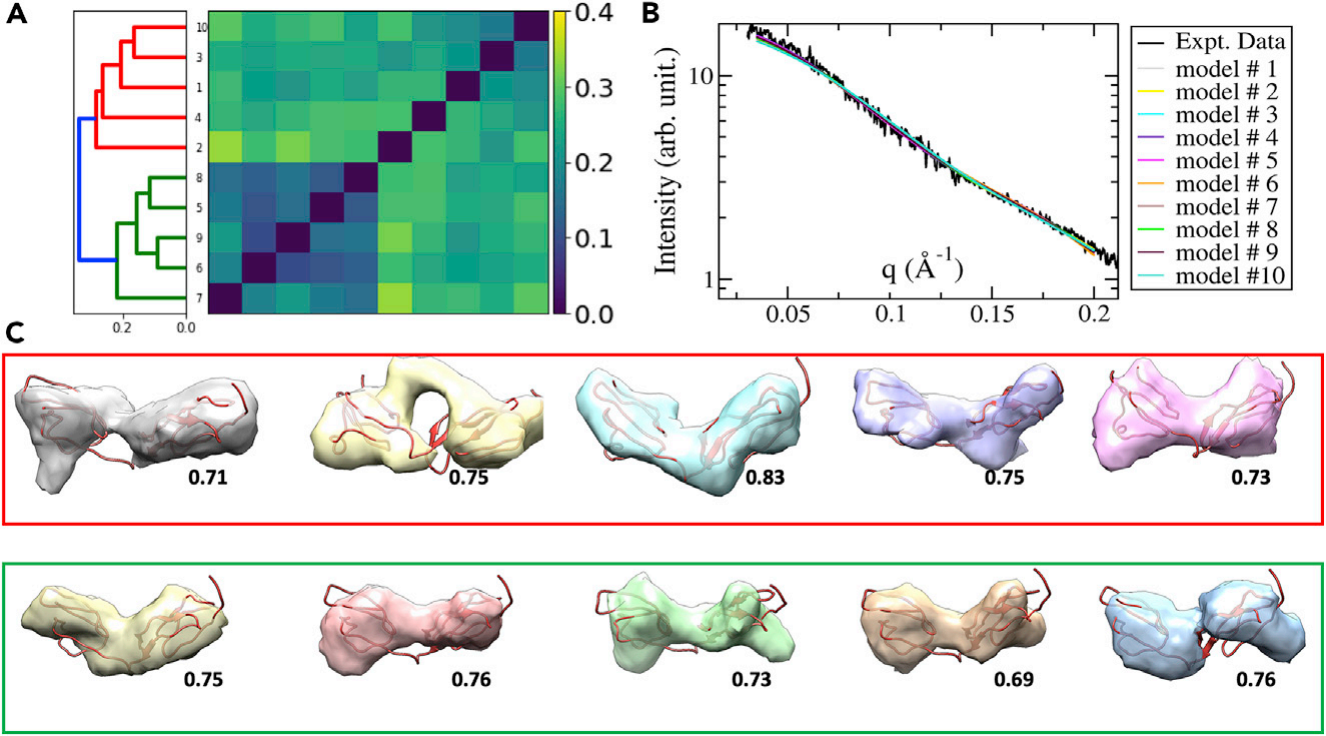
Reconstruction Model Quality and Consistency (A) The clustering analysis of 10 reconstructed models for dataset SASDA25. Using the correlation distance cutoff of 0.3, 10 reconstructed models were classified to two groups (see [C] for the reconstructed models superposed to the atomic structure of the same protein). (B) The theoretical SAXS profiles of reconstructed models were fitted to the experimental data. (C) The 10 reconstructed models grouped into two classes, enclosed by the red and green boxes, corresponding to the two classes in (A). The correlation coefficients compared with the PDB structure are indicated next to each reconstructed model.

Because of the random initialization of the genetic algorithm, multiple reconstructions for the same SAXS dataset may lead to different models. Furthermore, the limited information contents in 1D SAXS profiles may also result in multiple 3D models that match to the experimental SAXS profile equally well. To test the model consistency, eight SAXS datasets were randomly chosen for multiple reconstructions. For each SAXS dataset, ten models were reconstructed by running the programs ten times with different initial models. Then the hierarchical clustering analyses were carried out using the correlation derived distance (see Transparent Methods). The clustering results indicate that single clusters were obtained in six of eight cases (see Table S3 for the clustering plots and the distance matrices). In the other two cases, the ten models were clustered into two classes (Figure 4A, as an example for dataset SASDBD:SASDA25). The computed model profiles in Figure 4B suggest similar levels of agreement to the experimental SAXS data. The reconstructed models for dataset SASDA25 are shown in Figure 4C, each superposed to the high-resolution PDB model. The models are grouped and enclosed using red and green boxes to indicate their classifications. The visual comparison and computed correlation coefficients compared with the PDB model both indicate that reconstructions are consistent with the reference PDB model (with *cc* > 0.70).

## Discussions and Conclusion

3D model reconstruction from 1D SAXS data is challenging given the limited information embedded in the 1D profile. Prior knowledge, especially size information, has been required for model reconstruction. Here, using the deep learning method, the 3D models can be compressively represented using 200-dimension vectors. Based on the success in dimension reduction using the auto-encoder neural network methods, a model reconstruction method is implemented by optimizing parameters in the 200-d latent parameter space. More importantly, this new method does not require model size information and demonstrates its robustness in 3D model reconstructions. Currently, the 200-d vector and the SAXS profile are not directly related but related via the decoding part of the neural network to get the corresponding 3D model and subsequent calculate its SAXS profile. This work demonstrates that the deep learning methods have potential applications in the interpretation of X-ray data, with properly designed interfaces between the model and X-ray data. The calculation speed may not have clear advantages over the methods that construct 3D models in real space, owing to the extra decoding procedure. Nevertheless, the proposed method opens up a new avenue for model reconstruction. It is possible to improve the efficiency and accuracy of model reconstructions, by expanding the approach presented in this work. An ultimate method is to compress the latent space to match the SAXS data, so that the trained neural network can directly “translate” the SAXS information to 3D models. In such frameworks, there are information inputs from both 3D models and 1D SAXS data, and to train such neural networks, more complicated neural network architecture is desired. In a separate study, preliminary results show the possibility to encode a SAXS profile using low-dimensional vectors rather than computing from the decoded 3D models, and this feature will significantly reduce the computing time. Linking the 200-D parameter in this study and the latent space vector for SAXS data encoding will be another alternative toward a *de novo* model reconstruction method without using iterative model building. On the other hand, the present approach might be slow, but it can be easily expanded to other applications with an interface to convert decoded 3D models to experimental measurable information (SAXS profile in this case).

Neural network training and application can benefit from parallel processing of GPU; the speed gained from advanced hardware can facilitate the multiple reconstructions for model consistency examination. The computing time for model reconstruction is not the bottleneck in SAXS studies, considering that the sample preparation and experimental execution usually take much longer time. Furthermore, the auto-encoder is not limited to represent uniform density models; therefore, it is possible to expand the neural network to encode models with density variations from the uniform density approximation models.

The progression of latent space parameters showed large variations of parameter values at the beginning stage of the optimization, and these parameters were converged to fixed values when the optimization progressed to 60–80 iterations. The linear interpolation between initial random values and the final optimized values can be decoded to 3D models as “intermediate models.” The interpolation of latent space parameters resulted a morphing of the decoded 3D models, from a roundish object to a model that matches SAXS data, revealing the relation between latent space parameters and real space models (see Video S2 for a demonstration of model morphing using latent parameter interpolation).

Video S2. Video Demonstration of “Intermediate Models” by Interpolate Latent Space Parameters, Related to Figure 2 The interpolated values of latent space parameters are obtained with linear combinations of initial parameters (randomly sampled) and the final parameters, by varying the weighting factors. This is for the reconstruction of SASDA38.

As the first demonstration for the deep learning method applied in the 3D model reconstruction from SAXS data, this work may open doors for potential applications of deep learning in understanding other types of experimental data, especially those that are insufficient to be directly converted to 3D models. For instance, imaging experimental data with one or a few 2D projection or scattering information could be analyzed in a similar approach to build 3D models. This method has demonstrated its capacity and robustness in building 3D models with diverse shapes from experimental data. As more high-throughput X-ray data are being collected, we anticipate increasing applications of such methods in data analysis.

### Limitations of Study

Owing to the low information content embedded in SAXS data, the present method is limited to the reconstruction of models with uniform density. In principle, the method can be expanded to reconstruct higher-resolution models to reflect density variations within molecular envelopes. This shall require more advanced neural network architecture to encode models with density variations.

The maximum scattering angle used for model reconstruction is 0.2 Å^−1^ in this study. Proper modeling of the hydration layer at molecular surfaces is desired to utilize the scattering signals at larger scattering angles.

## Methods

All methods can be found in the accompanying Transparent Methods supplemental file.

## Data and Code Availability

The data and program codes are available at http://liulab.csrc.ac.cn/decodeSAXS or https://github.com/LiuLab-CSRC/SAXS_reconstruction.

## Supplemental Information

Document S1. Transparent Methods, Figures S1–S4, and Tables S1–S3

Table S4. The Experimental SAXS Dataset Information, Related to Figure 1 The dataset is composed of experimental SAXS data with deposited models: 506 from SASDBD and 36 from Bioisis databases.

## Footnotes

Supplemental Information can be found online at https://doi.org/10.1016/j.isci.2020.100906.
