# Supplementary material for: Model Reconstruction from Small-Angle X-Ray Scattering Data Using Deep Learning Methods
Source: iScience. 2020 Feb 13;23(3):100906. doi: 10.1016/j.isci.2020.100906 (PMC7037568; doi:10.1016/j.isci.2020.100906)
Supplement: Document S1. Transparent Methods, Figures S1–S4, and Tables S1–S3 [file mmc1.pdf]

iScience, Volume *23*

Supplemental Information

Model Reconstruction from Small-Angle X-Ray Scattering Data Using Deep Learning Methods

Hao He, Can Liu, and Haiguang Liu

Table S1. The detailed configuration for each layer, related to Figure 1b.

layer	parameters	shape
Input:		In:(32,32,32,1)
conv1_1	k= (3,3,3,1,64) strides = (1,1,1,1,1)<br>padding ="SAME "	In:(32,32,32,1)<br>out:(32,32,32,64)
conv1_2	filter = (3,3,3,64,64) strides = (1,1,1,1,1)<br>padding = "SAME "	In:(32,32,32,64)<br>Out:(32,32,32,64)
relu		
max_pool1	ksize = (1,2,2,2,1)<br>strides = (1,2,2,2,1)	In:(32,32,32,64)<br>out:(16,16,16,64)
conv2_1	filter = (3,3,3,64,128) strides = (1,1,1,1,1)<br>padding ="SAME "	In:(16,16,16,64)<br>out:(16,16,16,128)
conv2_2	filter = (3,3,3,128,128) strides = (1,1,1,1,1)<br>padding ="SAME "	In:(16,16,16,128)<br>out:(16,16,16,128)
relu		
max_pool2	ksize = (1,2,2,2,1)<br>strides = (1,2,2,2,1)	In:(16,16,16,128)<br>out:(8,8,8,128)
conv3_1	filter = (3,3,3,128,128) strides = (1,1,1,1,1)<br>padding ="SAME "	In:(8,8,8,128)<br>out:(8,8,8,128)
conv3_2	filter = (3,3,3,128,128) strides = (1,1,1,1,1)<br>padding ="SAME "	In:(8,8,8,128)<br>out:(8,8,8,128)
conv3_3	filter = (3,3,3,128,128) strides = (1,1,1,1,1)<br>padding ="SAME "	In:(8,8,8,128)<br>out:(8,8,8,128)
relu		
fc1		In:(8,8,8,128)<br>Out:(200)
relu		
fc2		In:(200)<br>Out:(8,8,8,128)
relu		
deconv1	filter = (5,5,5,64,32) strides = (1,2,2,2,1)<br>padding ="SAME "	in:(8,8,8,32)<br>out:(16,16,16,64)
relu		
deconv2	filter = (5,5,5,128,64) strides = (1,2,2,2,1)<br>padding ="SAME "	in:(16,16,16,64)<br>out:(32,32,32,128)
relu		
conv4	filter = (3,3,3,128,1) strides = (1,1,1,1,1)<br>padding ="SAME "	In:(32,32,32,128)<br>out:(32,32,32,1)
sigmoid		

conv: convolution layer; **fc**: fully-connected laser; **max_pool**: pooling layer; **relu**: activation operator; **deconv**: deconvolution layer.
Gray shaded layers are for the encoder part, and the blue shaded layers are for the decoder part, respectively.

The Tensorflow framework is used for the neural network implementation and training.

The encoder part is composed of seven convolutional layers and two pooling layers, connected with Relu activation functions, and the last layer of the encoder part is a fully connected layer.

The decoder part includes a fully connected layer, followed by two cov3d_transpose layers, and concluded with a conv3d layer. It also utilizes Relu activation functions between layers and a sigmoid function to obtain the final output.

In the auto-encoder network, Relu activation function is applied after conv1_2, conv2_2, conv3_3, fc1, fc2, deconv1, deconv2. The pooling method used in the encode part is max-pooling with padding, and the stride is 2 in 3d space.

The decoder part utilizes conv3d_transpose with padding to increase its size, and the stride is 2 in 3d space.

Key parameters used for training:

BATCH_SIZE = 64
LEARNING_RATE = 0.01 , 0.001 or exponential_decay
TRAINING_EPOCHS = 15

The optimizer function is AdamOptimizer, and the cross entropy was used as the loss function.

Two-stage network training

The auto-encoder network model was trained in two stages, with shapes in the PISA database as the training dataset, which contains 60,000 samples.

Stage-1:

Setting the linking layer between encoder and decoder (i.e., the fc1-output and fc2-input, or the bottleneck layer) to be 3,000 (i.e., the 3D models are encoded to 3,000-D vectors), and other parameters are listed in Table S1. When the training result converges, the preliminary auto-encoder model is saved for the second stage training.

Stage-2:

Based on the auto-encoder model trained in Stage-1, the linking layer size is reduced to 200, and re-train the fc1 and fc2 layers **only**, while retaining other parameters in the rest of the auto-encoder network. The final trained network will have 200 parameters in this linking layer for 3D shapes.

Alternatively, the training can also be accomplished with a single stage training network by inserting the 200d vector layer to the first stage model, right after the layer of 3000 variables. These two layers are linked using a fully connected network. The two approaches yielded similar results. The source codes for both approaches are available from the Github repository.

Table S2. Representative reconstructed models compared to the reference models, related to Figure 2.

*Rref: Radius of reference model deposited to the database (in the unit of Å)

#Ropt: Radius optimized using the genetic algorithm in the *decodeSAXS* program (in the unit of Å)

To illustrate the model reconstruction accuracy at various correlation coefficient (*cc*) levels (in ascending order), reconstructed models are superimposed to the models deposited to the database. The deposited models are either coarse grain bead models or high resolution atomic models.

Based on visual comparison, the model quality is very good if the cc is greater than 0.70. For the models with low cc values, major causes are that the protein complexes are very dynamic and flexible, or loosely packed (see SASDB47, SASDB98). For SASDCW6, the SAXS data were collected with a buffer matching method to mask the histone protein component, so that the DNA superhelices were the only 'visible' component for X-rays. This is very unusual for biomolecules, so the *decodeSAXS* failed in 3D model reconstructions for this dataset. For SASDC47, the optimized radius (Rmax) was wrong, resulting a wrong model.

As shown in the main text, using cc=0.70 as a threshold, about 54% reconstructions are in good quality; if the cc threshold is relaxed to 0.55 (average *cc* for randomly paired models) in this dataset, then about 86% reconstructions can be considered to be successful. The performance does not rely on the prior knowledge of the molecular sizes, and this is a unique feature of this method.

Data ID	*cc*	Rref*/ Ropt#	View-1	View-2	View-3	Fitting I(q)
SASDC 47	0.14	59.19/ 85.90	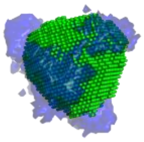	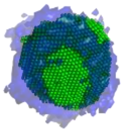	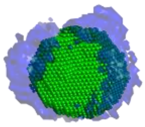	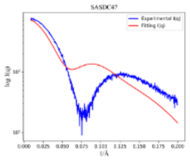
SASDB D6	0.41	53.72/ 64.60	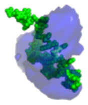	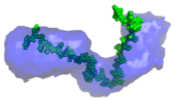	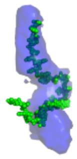	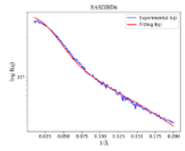
SASDC W6	0.43	88.72/ 111.85	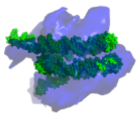	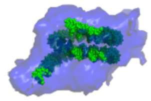	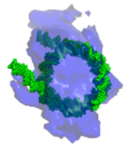	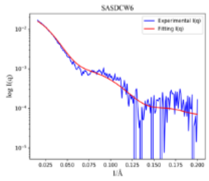
SASDC Y7	0.45	57.17/ 84.45	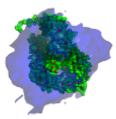	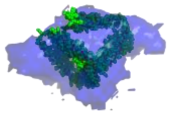	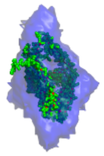	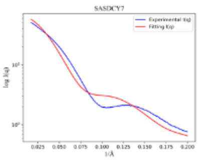
SASDB 47	0.47	84.38/ 103.05	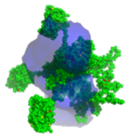	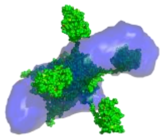	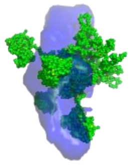	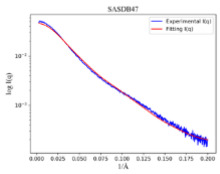

Data ID	*cc*	Rref*/ Ropt#	View-1	View-2	View-3	Fitting I(q)
SASDB 98	0.48	70.60/ 89.30	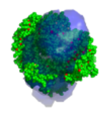	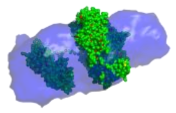	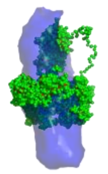	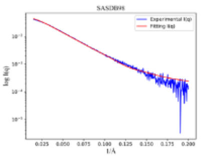
SASDA W8	0.57	70.78/ 78.25	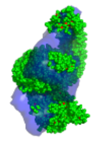	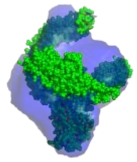	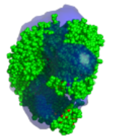	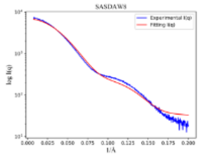
SASDA P5	0.67	48.58/ 58.60	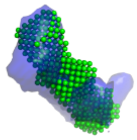	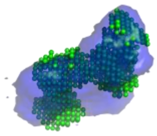	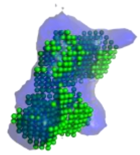	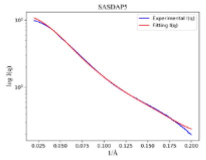
SASDA V5	0.68	75.21/ 78.50	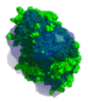	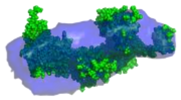	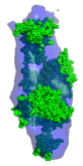	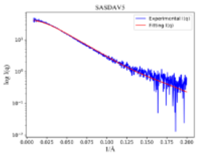
SASDA R5	0.71	40.35/ 52.05	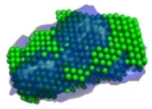	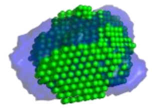	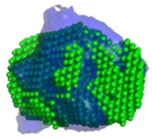	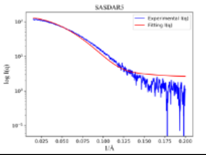
SASDB 46	0.73	74.51/ 70.25	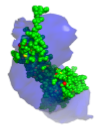	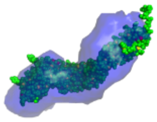	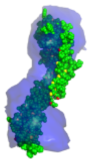	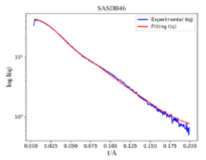
SASDA Q7	0.79	87.67/ 90.65	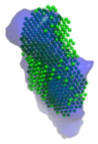	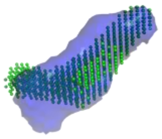	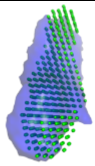	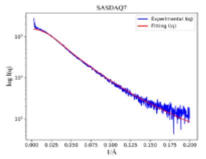
SASDD F9	0.80	35.94/ 51.95	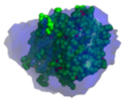	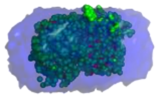	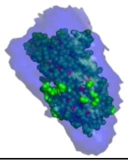	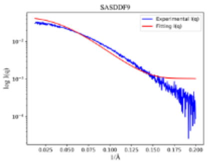
SASDA P8	0.84	69.74/ 77.85	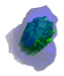	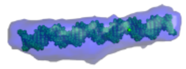	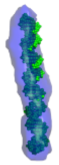	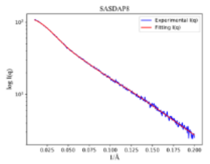

Data ID	*cc*	Rref*/ Ropt#	View-1	View-2	View-3	Fitting I(q)
SASDC G5	0.85	24.63/ 27.75	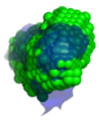	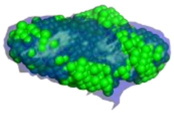	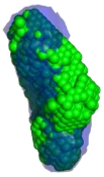	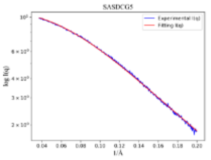
SASDB Y3	0.85	82.28/ 69.10	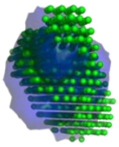	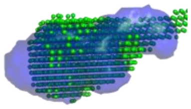	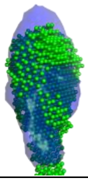	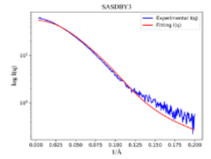
SASDA N8	0.87	85.90/ 82.05	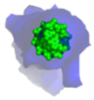	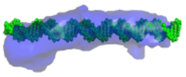	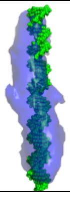	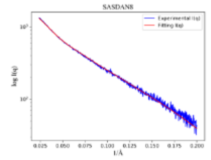
SASDA U5	0.87	83.48/ 82.30	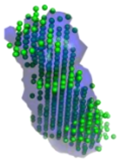	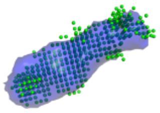	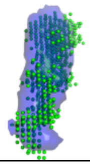	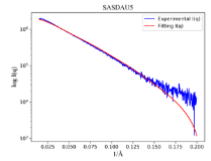
SASDB E2	0.87	46.95/ 53.55	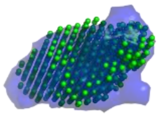	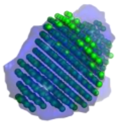	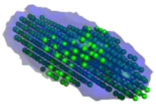	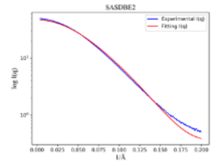
SASDB 32	0.88	37.37/ 39.00	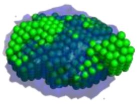	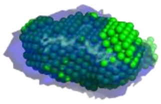	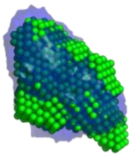	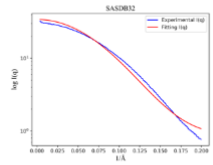
SASDB Q3	0.91	48.44/ 46.15	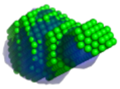	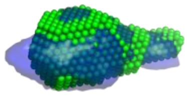	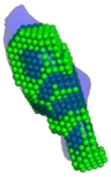	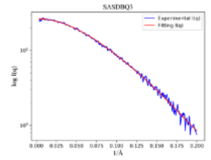
SASDB X4	0.93	101.57 /95.60	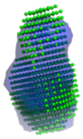	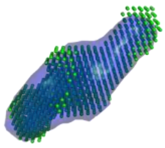	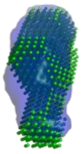	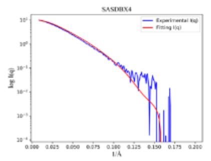

Table S3. Hierarchical clustering analysis of multiple reconstructions for 8 tested datasets, related to Figure 4.

Eight SAXS datasets were randomly selected for multiple reconstruction tests. The correlation between reconstructed models were computed. The hierarchical clustering method was applied using the distance metric defined as d=1.0 – cc, where cc is the correlation coefficient between reconstructed models calculated using sastb.superpose. Using d=0.3 as cutoff (i.e., correlation coefficient=0.7), 6 out of 8 testing cases showed single cluster; and 2 cases (SASDA25 & SASDA28) showed two clusters.

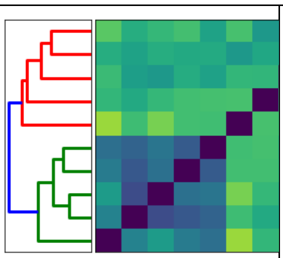	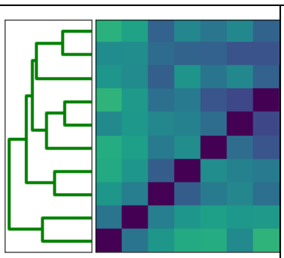	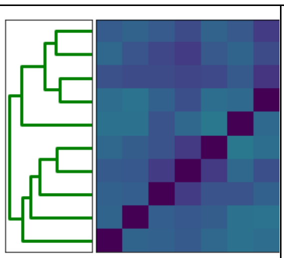	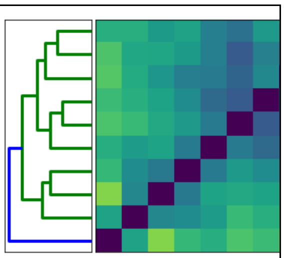
**SASDA25**	**SASDA26**	**SASDA27**	**SASDA28**
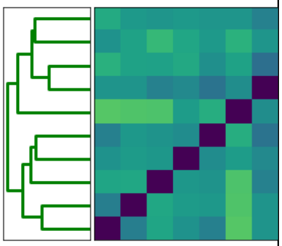	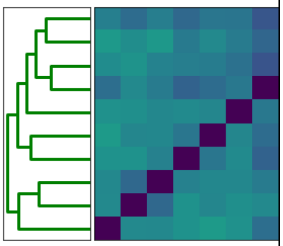	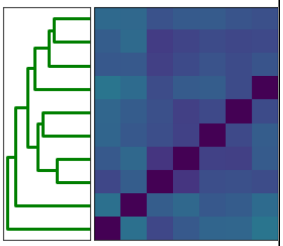	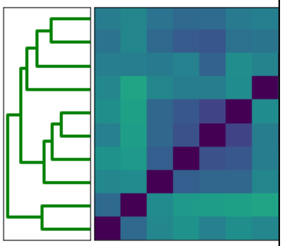
**SASDA32**	**SASDA35**	**SASDA38**	**SASDA39**

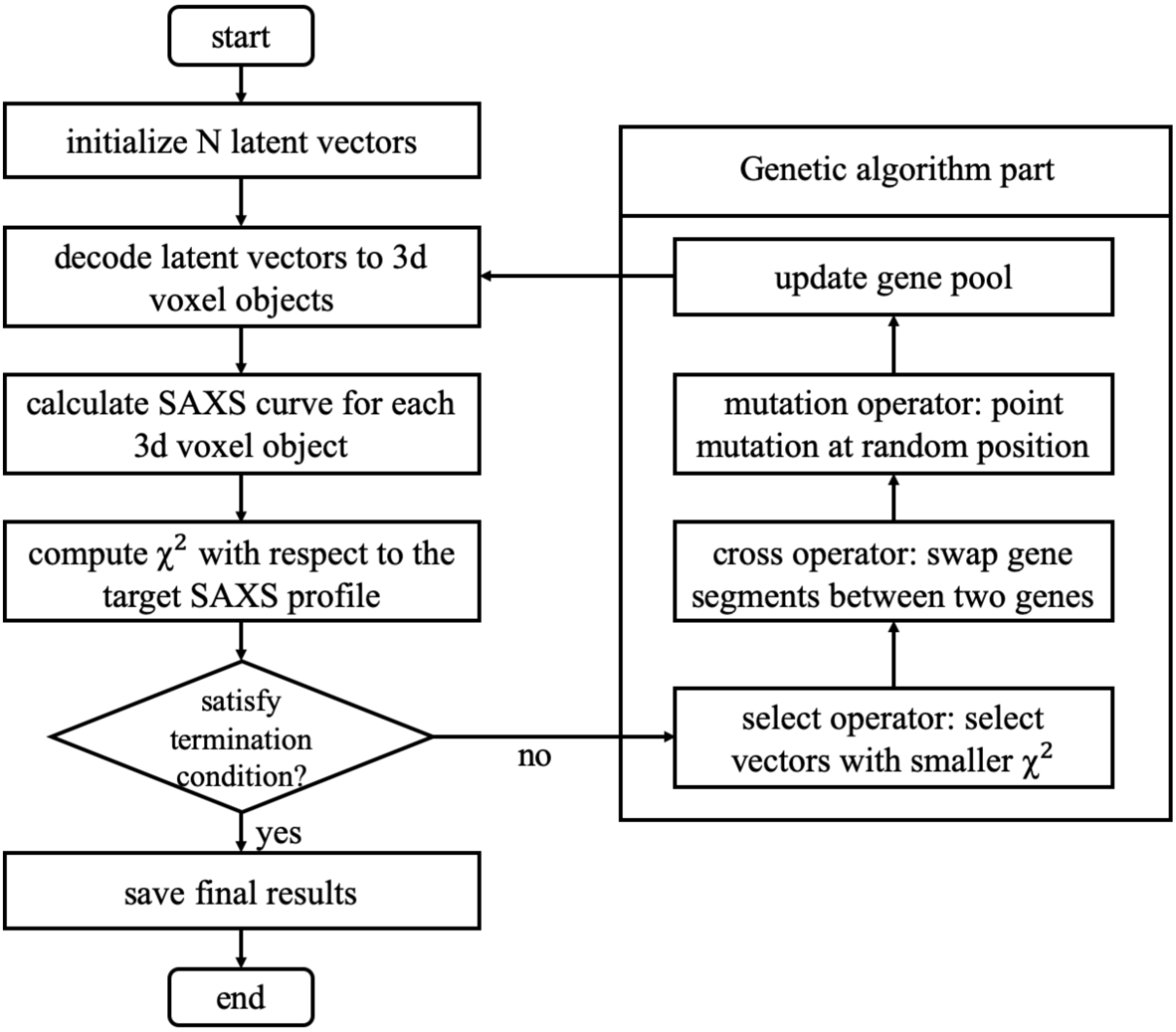

Figure S1. The flowchart of model reconstruction algorithm, related to Figure 2. The auto-encoder-decoder neural network is used to decode latent space parameter to 3D voxel models, whose SAXS profiles are compared to experimental data. Genetic algorithm is used to guide the optimization of latent space parameters.

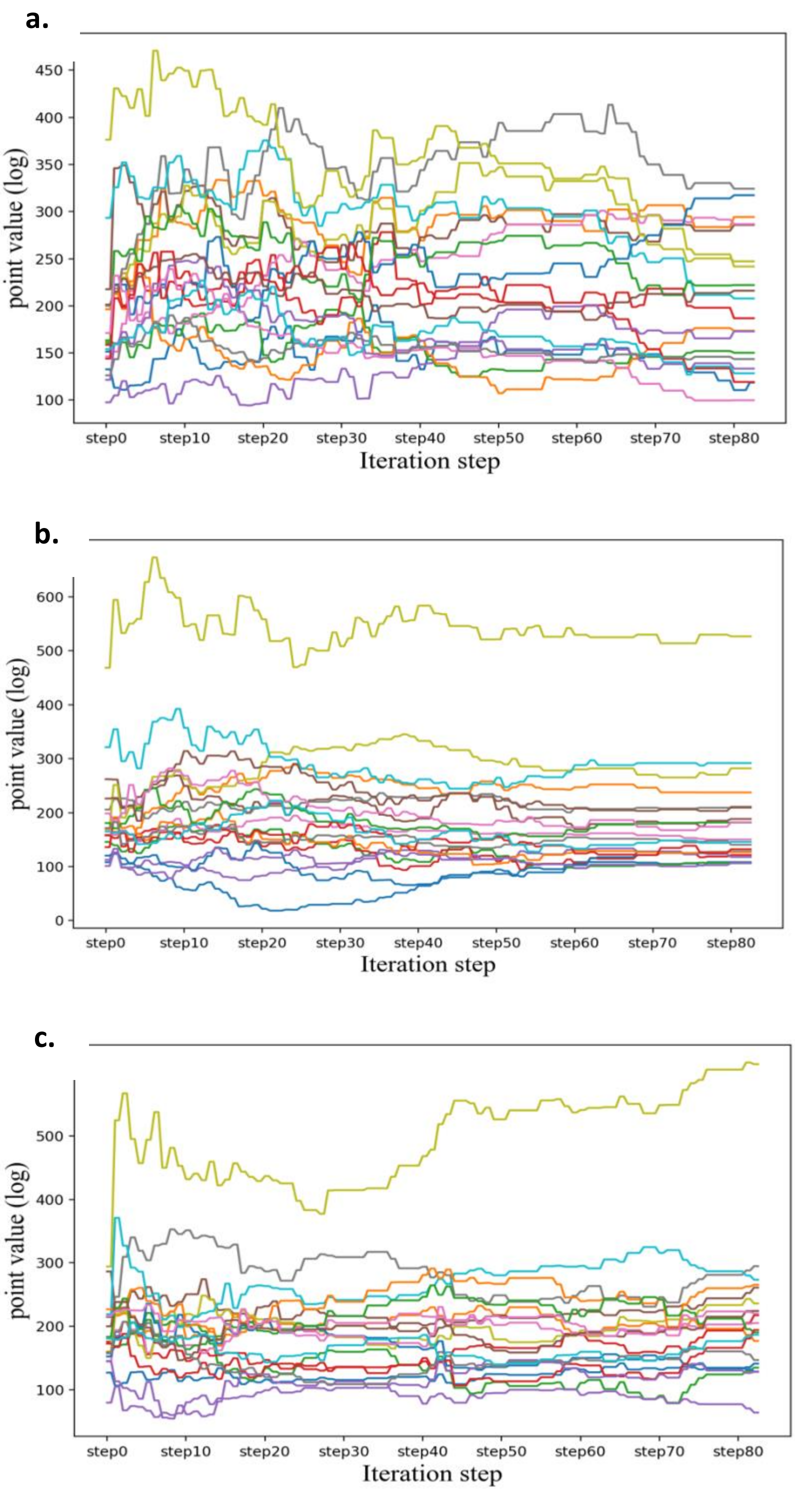

Figure S2. The progression of the first 20 parameters in the latent space, related to Figure 2. The parameters widely vary at the beginning and the convergence start to emerge after some iterations. Three examples are shown as (**a**) SASDA38; (**b**) SASDBQ3; and (**c**) SASDBY3.

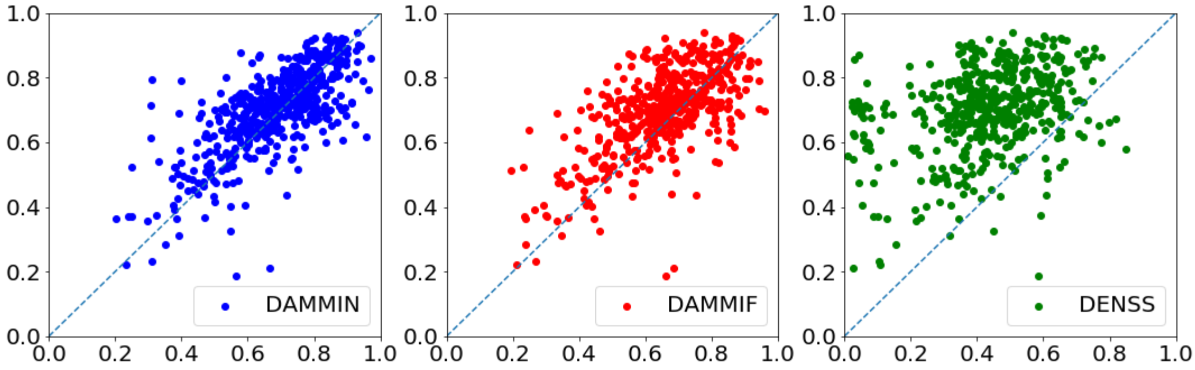

Figure S3. The performance comparison between decodeSAXS and three other methods, related to Figure 3. The scatter plots are shown for the cc values: y-axis shows the cc values between reference models and the models reconstructed using decodeSAXS; x-axis shows the cc values between reference models and the models reconstructed using the three other methods (labelled in the legends). The points above the line (y=x) corresponding to the cases that decodeSAXS performs better.

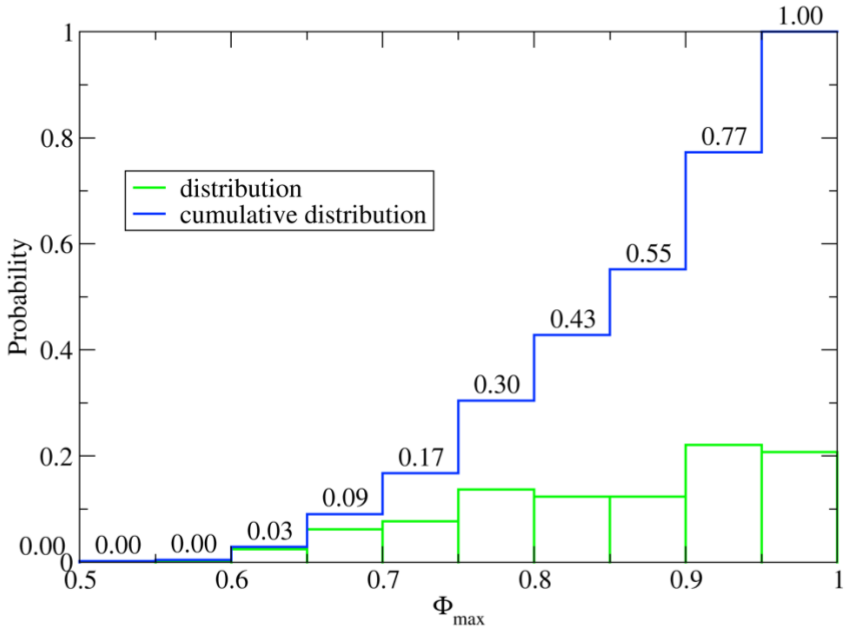

Figure S4. The distribution of Phi$_{max}$ values, related to Figure 2. The values are computed between the reconstructed models with decodeSAXS and the reference models. The distribution (green histogram) shows that Phi$_{max}$ has a larger population at larger values, compared to the cc values (Figure 2d). The cumulative distribution (blue line) shows that 70% of the Phi$_{max}$ values are larger than 0.80.

Transparent Methods

Training and Testing Datasets

The model dataset is compiled from the PISA structure database, including 60,000 randomly selected 3D models. Each model was first scaled and shifted to fit in a sphere centred at the coordinate origin with a radius of 50 Å (the shape does not depend on the size of the model under the uniform density model approximation; the size information is used for SAXS profile computation, see equation 2 below). Then, the atomic positions were mapped to a grid of 31x31x31 in the process of scaling and voxelization (the grid point at (0,0,0) coincides with the center of mass). As a result, each model was converted to a voxel object described using a 3D matrix with binary values (i.e., the uniform density is ensured, see Figure 1a in main text). For numerical calculation efficiency consideration, the matrix of 31x31x31 was padded with zeros to a matrix of 32x32x32, such that the number of discretization is a power of 2, to facilitate the convolutional neural network training.

Auto-encoder Neural network architecture

The architecture of the auto-encoder is designed based on the VGG network (Simonyan and Zisserman, 2015). The encoding part of the auto-encoder is composed of seven convolution layers and two pooling layers followed by one dense (fully connected) layer as indicated in Figure 1b and described in Table S1. Network training was performed in two stages. During the first stage, the dense layer contains 3,000 variables, and this number is reduced to 200 during the second stage. With this design, the 3D shape information is compressed to a representation of 3,000-dimensional vectors after the first training stage. Among these 3,000 parameters, a significant portion (approximately 90%) of parameters is found to be zero persistently, indicating that the parameter space for encoding can be further reduced. With this observation, the fully connected layer was optimized again with a reduced dense layer of 200 parameters. During the second stage of training, the parameters for convolutional and pooling layers were inherited from the first stage and remained unchanged, except that the parameters for the fully connected dense layer were subjected to optimization. This two-stage training was adapted to ensure fast convergence of the training (we found that if the dense layer was set to a 200-dimension vector during the first stage of training, the loss function does not converge). There is an alternative architecture to allow the training to be completed within a single stage, by adding the 200-d layer after the 3000-d layer and linked with fully connected network. The two approaches yield similar results (the network architectures and training codes are available at the Github repository). Similar to many cases in neural network training/applications, the choice of 200 parameters for this compressed layer is not unique. However, the encoding capacity will be affected if fewer parameters were used.

The decoding part is relatively simple. The 200-dimension vector is converted to a 4D tensor of size 8x8x8x32, then followed by two deconvolution layers, and finished with a convolution layer to obtain a 32x32x32 matrix, from which a submatrix of 31x31x31 was obtained as the reconstructed model. A preset threshold of 0.1 was used to convert the matrix to binary values.

The 60,000 models in the training dataset were fed to this auto-encoder with the loss function measured by cross entropy between the input models and the encode-decoded maps. The decoding part of the neural network can be applied to interpret any 200-d vectors to its corresponding 3D density map in the form of voxel object.

Model reconstruction from SAXS profiles

The overall workflow for model reconstruction using the auto-encoder method is as follows (see Figure S1). First, a number of latent parameter sets (or genes, in terms of genetic algorithm) are generated to initialize the genetic population to be optimized by genetic algorithm. The parameters for each gene is sampled based on the gene value distribution at the corresponding positions (see Figure 1d for representative probability distributions). After initialization, the iterative optimizations will be carried out. During each iteration, the genes are decoded to 3D voxel objects using the auto-encoder network trained using the molecular shapes abstracted from the PISA database. To ensure the continuity of the reconstructed models, only the largest connected domain of each decoded object will be kept as the ‘cleaned model’. The SAXS profiles for the ‘cleaned models’ are then computed using the Zernike expansion method implemented in the SASTBX (elaborated below). Chi-scores between model profiles and target SAXS profiles are used to guide the genetic algorithm to evolve the genes until the chi-score is converged or a pre-set number of iterations is reached. The final models are saved in density maps (CCP4 format) or bead models (PDB format).

For a voxel object, the 3D Zernike representation has the advantage of de-coupling the model shape and size information; thus, the SAXS profiles can be quickly evaluated if the model size is updated while the shapes are not changed. The detailed derivation was elaborated elsewhere (Liu et al., 2012a, 2012b), and a brief summary is provided for clarity. A 3D object $r(\mathbf{r})$ after scaling to fit within a unit sphere can be expanded as the weighted summation of 3D Zernike functions, which are a set of orthonormal polynomials with orders (*n, l, m*) (Canterakis, 1999; Novotni and Klein, 2003). The expansion coefficient or the so-called Zernike moment C_{nlm} at order (*n, l, m*) is calculated with equation (1):

$$C_{nlm} = \int_{|\boldsymbol{r}|<1} \rho(\boldsymbol{r}) Z_{nlm}(\boldsymbol{r}) d\boldsymbol{r} \tag{1}$$

Subsequently, the 3D density distribution function $r(\mathbf{r})$ can be approximated using $\{C_{nlm}\}$ up to a maximum expansion order $n_{\max}$. It has been shown (see (Liu et al., 2012a)) that the SAXS intensity can be explicitly expressed as:

$$I(q) = \sum_{n} \sum_{n'} B_n(qr_{\max}) B_{n'}(qr_{\max}) H_{nn'} \tag{2}$$

where $B_n(qr_{\max}) = \frac{j_n(qr_{\max}) + j_{n+2}(qr_{\max})}{2n+3}$ describes the contribution from corresponding Zernike polynomials to SAXS profile with the Bessel functions of the first kind $j_n(x)$. In addition, the shape information is encoded in $H_{nn'}$, which is expressed using Zernike moments as follows:

$$H_{nn'} = \sum_l k_{nl} k_{n'l} \sum_m C_{nlm} C^*_{n'lm} \tag{3}$$

with $k_{nl} = (-1)^{\frac{n-l}{2}}$. According to equation (2), the radius of the object, r_{max}, is readily decoupled from the shape descriptors {$H_{nn'}$}. This allows us to optimize the radius as the 201st parameter (the other 200 parameters encode the 3D shape). The SAXS profiles can be calculated with other programs, as long as the program can be interfaced to the reconstruction software by providing the calculated intensities at desired scattering angles. In the case if the radius optimization is desired, the SAXS profile calculation is slightly more complicated, because the model scaling might be needed.

The genetic algorithm was used to optimize the parameters (200 parameters for given radius information or 201 parameters if the radius is a free parameter to be optimized) (Goldberg, 1989). The target function to be minimized is the standard chi-score:

$$\chi^2 = \frac{1}{N} \sum_{i=1}^{N} \left[\frac{I_i^{\exp} - I_i^{\mathrm{mod}}}{\sigma_i} \right]^2 \tag{4}$$

Where $I_i^{\exp}$ and I_i^{mod} are the SAXS intensity profiles of the experimental measurement and the model calculation respectively, both at position q_i. The SAXS profiles were normalized to the range of [0,1] before the chi-score computation to remove the mismatched scaling factor and the offset level that caused by the residual background intensity. The $1/\sigma_i$ was used as the weighting factor. It is noted that the raw SAXS data were preprocessed using a sliding polynomial fitting (with order=2, window size=5) to smooth the profile. In order to balance the contributions from all data points to the chi-score, we compared three different weighting schemes, specifically, setting σ_i to 1.0, sqrt(I_i^{exp}), and I_i^{exp}. The results suggested that the final reconstructed models reached similar accuracy levels. In the program, the default weighting scheme is σ_i =1.0. It is noteworthy to point out that chi-score is just one of many possible metrics to quantify the difference between model profile and experimental data. Other formulations, such as likelihood functions based on probability theory can be also adapted easily by replacing the scoring function in the program. In this study, the testing results showed that the present implementation can achieve very good model quality.

The distribution of latent parameter values obtained during the auto-encoder training procedure was used to guide the initialization of model parameters, so that the genes (each with 200 or 201 values) for the first generation of the genetic algorithm were populated by sampling the latent parameter distributions to start the optimization procedure. In each generation, 300 genes are generated via simulated evolution procedures. The gene evolution was implemented using three operators:

selection, crossing, and mutation. The selection operator decides which genes are inherited from the previous generation by selecting the more fitted gene out of two randomly selected genes. The crossing operator is included to exchange gene segments obtained from the previous generation. The mutation operator changes parameter values at random positions by replacing the current value to a random value with a probability distribution that follows prior knowledge of empirical distributions at that gene position.

The model comparison was measured using the correlation coefficients (*cc*) after model alignment to the reference model (not used during model reconstruction process), which was performed using the fast rotation algorithm implemented in sastbx.superpose (Liu et al., 2012b). The correlation coefficient used to compare variables with binary values is referred to as Phi coefficient, similar to the pearson correlation coefficient for variables with continuous values (Guilford, 1936). In statistical analysis, the range for Phi coefficient is not strictly confined in [-1,1] (Davenport and El-Sanhurry, 1991). We computed the Phi_{max} between decodeSAXS reconstructed models and the reference models to provide a reference (the values are mostly above 0.80, see Figure S4 for a distribution). Here, the concept of Phi coefficient is borrowed to measure the similarity between reconstructed model and the reference model. The computed *cc* is used to quantify the overlapped volume from the two models being compared. The physical interpretation is the that *cc*=1.0 corresponds to perfect overlapping (aligned to itself), and *cc*=0.0 corresponds to zero overlapping between the two models. Both Phi_{max} and cc can be used to measure the model similarity. The cc values are used to quantify the model similarity through the analysis because of its clear physical interpretation, and the Phi_{max} is provided as a reference. For consistency analysis for models from multiple reconstructions, the hierarchical clustering algorithm was applied using correlation distance (defined as 1.0-*cc*). The figures were prepared using Chimera (Pettersen et al., 2004) or Pymol (Schrödinger, 2015).

Supplemental Reference

Davenport, E.C., and El-Sanhurry, N.A. (1991). Phi/Phimax: Review and Synthesis. Educ. Psychol. Meas. *51*, 821–828.
Guilford, J.P. (1936). Psychometric methods, (New York; London: McGraw-Hill Book Company, Inc.).
Novotni, M., and Klein, R. (2003). 3D zernike descriptors for content based shape retrieval. In Proceedings of the Eighth ACM Symposium on Solid Modeling and Applications, (New York, NY, USA: ACM), pp. 216–225.
Pettersen, E.F., Goddard, T.D., Huang, C.C., Couch, G.S., Greenblatt, D.M., Meng, E.C., and Ferrin, T.E. (2004). UCSF Chimera - A visualization system for exploratory research and analysis. J. Comput. Chem. *25*, 1605–1612.
Schrödinger, L. (2015). The {PyMOL} Molecular Graphics System, Version~2.2.
